# *Enterobacter*-Activated Mosquito Immune Responses to *Plasmodium* Involve Activation of SRPN6 in *Anopheles stephensi*

**DOI:** 10.1371/journal.pone.0062937

**Published:** 2013-05-03

**Authors:** Abraham G. Eappen, Ryan C. Smith, Marcelo Jacobs-Lorena

**Affiliations:** Department of Molecular Microbiology and Immunology, Malaria Research Institute, Johns Hopkins Bloomberg School of Public Health, Baltimore, Maryland, United States of America; Museum National d’Histoire Naturelle, France

## Abstract

Successful development of *Plasmodium* in the mosquito is essential for the transmission of malaria. A major bottleneck in parasite numbers occurs during midgut invasion, partly as a consequence of the complex interactions between the endogenous microbiota and the mosquito immune response. We previously identified SRPN6 as an immune component which restricts *Plasmodium berghei* development in the mosquito. Here we demonstrate that SRPN6 is differentially activated by bacteria in *Anopheles stephensi*, but only when bacteria exposure occurs on the lumenal surface of the midgut epithelium. Our data indicate that *As*SRPN6 is strongly induced following exposure to *Enterobacter cloacae*, a common component of the mosquito midgut microbiota. We conclude that *As*SRPN6 is a vital component of the *E. cloacae*-mediated immune response that restricts *Plasmodium* development in the mosquito *An. stephensi*.

## Introduction

Malaria is among the deadliest infectious diseases, killing in excess of one million people every year, mostly of African children under the age of five. Transmission is entirely dependent on the completion of the life cycle of *Plasmodium*, the causative agent of malaria, in its mosquito vector. After ingestion of an infectious blood meal, *Plasmodium* gametocytes differentiate into male and female gametes that fertilize to generate a diploid zygote. After a round of DNA replication, the tetraploid zygote differentiates into a motile ookinete. At approximately 24 h after ingestion the ookinete invades the mosquito midgut and differentiates into sessile oocysts. Within 7 to 14 days (depending on parasite species), thousands of sporozoites are released from each oocyst into the mosquito hemocoel. Sporozoites must successfully invade the salivary glands to ensure transmission when the infected mosquito bites and inoculates sporozoites into a new individual [1], [2]. The parasite life cycle in its mosquito host is complex, and dramatic losses in parasite numbers occur at each stage of *Plasmodium* development [2], [3]. Ookinete midgut invasion represents the largest bottleneck in parasite numbers [2], [3], as ookinetes must overcome the effects of the mosquito midgut microbiota and the innate immune responses in order to successfully transition into an oocyst [4].

The mosquito midgut microbiota is very dynamic, with dramatic fluctuations based upon life-stage, nutritional status, and age [5]. After a blood meal, mosquito commensal bacteria undergo changes in their population structure to enrich for enteric gram-negative bacteria capable of surviving the harsh, digestive environment of the mosquito midgut [5]. Within this nutrient-rich environment, bacteria reach high numbers at a time that coincides with ookinete invasion (∼24 h post-blood meal) [6], and can greatly influence the success of *Plasmodium* parasite development [6]–[9]. In addition, the presence of endogenous bacteria is also thought to prime the mosquito innate immune response to limit parasite survival [9]–[11]. Basal expression levels of anti-microbial genes controlled by mosquito innate immune pathways limit bacterial proliferation and indirectly contribute to cross immune protection against *Plasmodium* parasites [9]–[12]. In the absence of midgut microbiota, mosquito susceptibility to *Plasmodium* infection is greatly increased [9].

While the involvement of the mosquito’s microbiota in the anti-*Plasmodium* response is beginning to be explored [8]–[12], the mosquito innate immune system also contributes a major role in parasite attrition [4], [13], [14]. As *Plasmodium* ookinetes reach the basal lamina of the midgut, parasites are subjected to components of the mosquito hemolymph that destroy a large proportion of the invading parasites [15]–[17]. Recent evidence suggests that parasite immune recognition is a critical determinant of invasion success, mediated by epithelial nitration of parasites during the process of midgut invasion [18].

Previously, we identified a putative serine protease inhibitor (SRPN6) that modulates rodent malaria parasite development in anopheline mosquitoes [19], [20]. In *An. stephensi*, SRPN6-silencing leads to a significant increase in *P. berghei* oocyst numbers [19]. However, in a susceptible line of *An. gambiae* (G3), SRPN6-silencing has no effect on the number of developing parasites [19]. Further experiments would suggest that *Ag*SRPN6 mediates parasite recognition and/or lysis and may additionally be involved in the regulation of the melanization response [19], [21], yet the precise function of SRPN6 in the mosquito immune response is still unknown.

In both *An. stephensi* and *An. gambiae*, SRPN6 is induced in response to *Plasmodium* ookinete invasion of the midgut [19], [22]. However, induction is more pronounced following *P. berghei* than *P. falciparum* infection with both mosquito species [19], [22]. The reasons for this difference remain unclear. Here we show that *As*SRPN6 can also be induced by certain bacteria, but only through contact with the lumenal surface of the midgut epithelium. Our experiments indicate that *E. cloacae* strongly induces *As*SRPN6 expression in the midgut shortly after feeding establishing this gene as an important component of the *E. cloacae*-mediated response that effectively inhibits *P. falciparum* development in *An. stephensi*.

## Results

### *As*SRPN6 Expression is Differentially Induced by Bacteria

**Figure 1:**
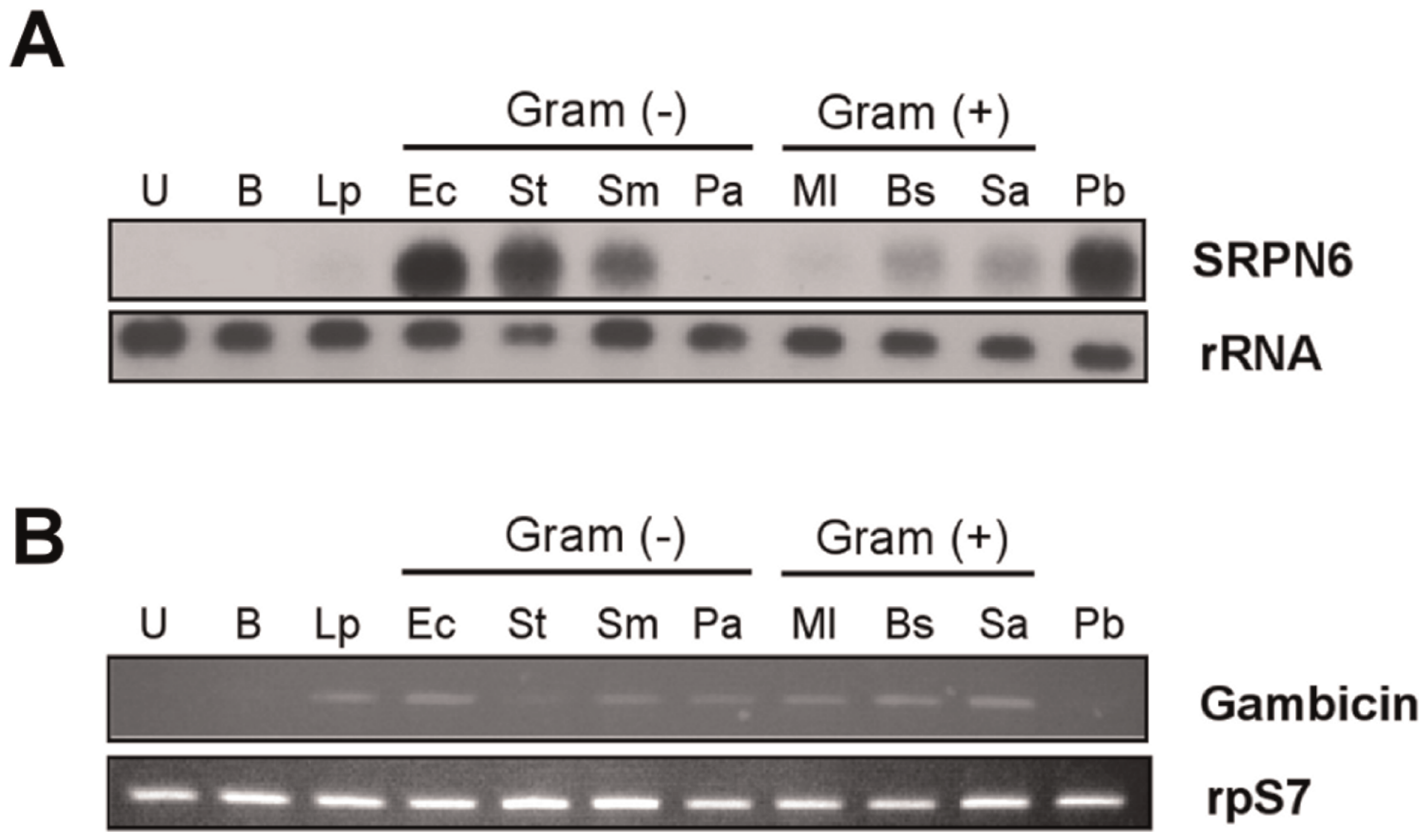
SRPN6 is differentially induced by bacteria in the mosquito midgut. (A) Bacteria (1×10^6^/ml of buffer; 2,000 bacteria assuming ingested volume of 2 µl) or the indicated component were fed to *An. stephensi* mosquitoes and their midguts were dissected 6 h later. Total RNA (3 µg) was analyzed by Northern blot using a ^32^P-labeled SRPN6 cDNA probe (upper panel). The blot was then stripped and hybridized with mitochondrial rRNA probe as a loading control (lower panel). Samples are identified above each lane as follows. U: unfed control; B: buffer-fed; Lp: *E. coli* LPS (10 mg/ml); Ec: *Enterobacter cloacae*; St: *Salmonella typhimurium*; Sm: *Serratia marcescens*; Pa: *Pseudomonas aeruginosa*; Ml: *Micrococcus luteus*; Bs: *Bacillus subtilis*; Sa: *Staphylococcus aureus*; Pb: *P. berghei*-infected blood, analyzed 24 h after feeding (positive control). (B) Expression of gambicin, a mosquito anti-microbial peptide. Gambicin transcript abundance was analyzed by semi-quantitative RT-PCR (upper panel) using ribosomal protein S7 (rpS7) mRNA expression as a loading control (lower panel). RNA templates are the same as those used in panel (A).

Previous experiments have demonstrated that SRPN6 expression is induced by *Plasmodium* ookinete invasion of the mosquito midgut [19], [22] and sporozoite invasion of the salivary glands [20]. To investigate whether bacteria also play a role in *As*SRPN6 activation, we measured changes in gene expression after feeding *An. stephensi* mosquitoes with different species of bacteria (Figure 1A). These experiments revealed that the intensity of *As*SRPN6 expression is differentially regulated by different bacterial species. *As*SRPN6 expression was strongly induced by most gram-negative bacteria examined, while gram-positive bacteria produced much weaker expression. These results agree with previous results that SRPN6 was primarily induced by gram-negative bacteria [9]. Interestingly, *As*SRPN6 expression was independent of the anti-microbial protein (AMP) gambicin [23], which is strongly expressed in the mosquito midgut and induced by most agents, including LPS and gram-positive bacteria but not by buffer or *P. berghei* parasites (Figure 1B). These observations suggest that multiple pathways may contribute to the midgut immune response to produce specific responses toward endogenous gram-negative bacteria and invading pathogens.

### *As*SPRN6 Expression Requires Signaling via the Lumenal Surface of the Midgut Epithelium

**Figure 2:**
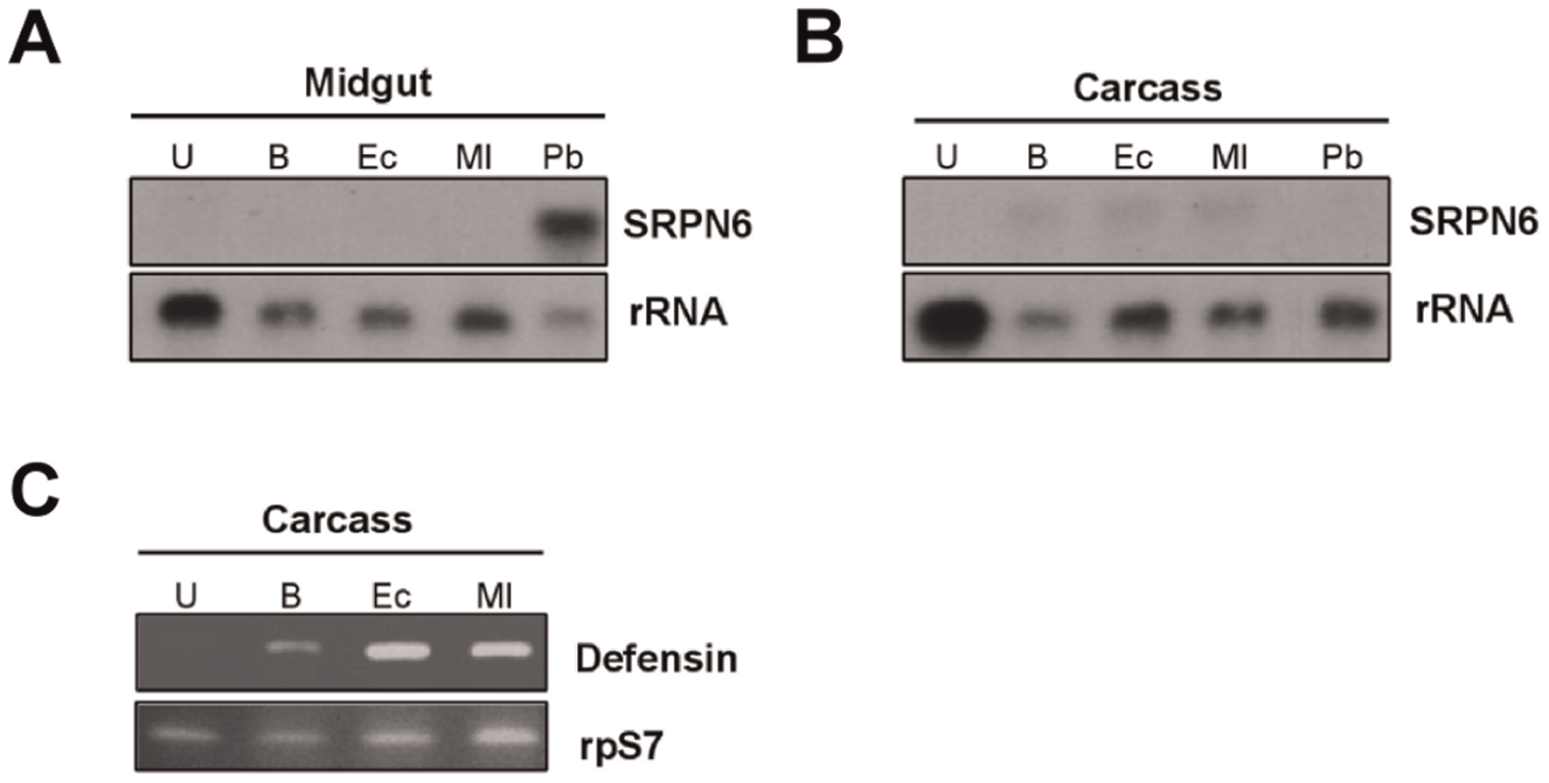
SRPN6 expression following bacterial injection into the mosquito hemocoel. Approximately 2×10^3^ bacteria were injected into the hemocoel of adult female *An. stephensi.* SRPN6 expression was analyzed by Northern blot in the midgut (A) and carcass (all non-gut tissues) (B) 6 h post bacteria injection of into the hemocoel. Procedures and abbreviations are the same as in Figure 1A. Similar results were obtained in three independent experiments. (C) As a control, expression of the anti-microbial peptide defensin was monitored by semi-quantitative RT-PCR in the carcass samples after bacteria injection. Procedures and abbreviations are the same as in Figure 1B.

The midgut epithelium consists of a single cell layer. On the lumenal side, epithelial cells display an extensive network of protrusions (microvilli), while the basal side is made of a complex invagination network (basal labyrinth) and a basal lamina exposed to the circulating hemolymph. The experiments outlined above examined *As*SRPN6 immune activation following bacteria exposure to the lumenal side of the epithelium via feeding. To investigate the signaling requirements needed for *As*SRPN6 activation, we sought to determine if bacterial exposure to the basal midgut surface was also capable of *As*SRPN6 induction. Following the injection of bacteria into the mosquito hemocoel, the levels of *As*SRPN6 expression were analyzed in midgut and carcass tissues (Figure 2). *As*SRPN6 transcript was undetectable in midgut samples following injection (Figure 2A), and only weakly detected in carcass samples (Figure 2B). These results suggest that activation of *As*SRPN6 midgut expression occurs via specific signaling mediated by the interaction of bacteria with the lumenal midgut surface.

As a control, carcass samples from bacteria-injected mosquitoes were also analyzed for defensin expression, a potent anti-microbial protein induced in the mosquito fat body [24]. While *As*SRPN6 and defensin were both weakly upregulated by injury (buffer injection), only defensin (and not *As*SRPN6) was strongly up-regulated by both *E. coli* and *M. luteus* (Figure 2C). These results suggest that the weak *As*SRPN6 expression in the carcass samples may be in response to injury and is independent of the presence of bacteria (Figure 2B).

### Different Patterns of *As*SRPN6 Induction by Bacteria and *P. berghei*

**Figure 3:**
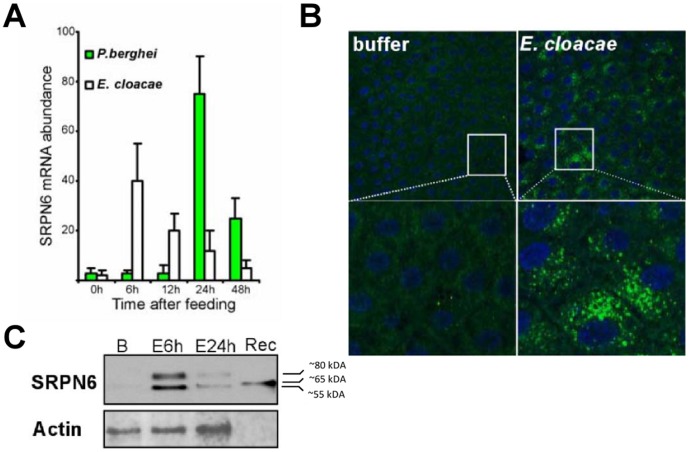
Activation of SRPN6 midgut expression by *E. cloacae*. (A) Time course of *An. stephensi* SRPN6 mRNA expression after feeding of *E. cloacae* (1×10^6^/ml; open bars) or *P. berghei* (green bars) as determined by qRT-PCR using ribosomal protein S7 (rpS7) for normalization. Values are reported in fold change relative to expression before feeding (0 h). (B) Immunolocalization of SRPN6 in midguts of mosquitoes fed with buffer (left panels) or *E. cloacae* (right panels). Guts were dissected 6 h after feeding, opened up into sheets, fixed and the protein detected with an anti-SRPN6 antibody. The inserts show higher magnifications of the areas within the squares. (C) Western blot analysis of SRPN6 protein expression after feeding with buffer alone (B) or at 6 and 24 h after *E. cloacae* ingestion, as indicated. Recombinant SRPN6 protein was used as a positive control (Rec). The blot was stripped and re-probed with an anti-actin antibody as a loading control (lower panel).

To better understand the mechanism of *As*SRPN6 induction by gram-negative bacteria, we compared the time course of *As*SRPN6 expression with that of *P. berghei,* which served as a positive control. Bacterial induction of *As*SRPN6 was rapid as mRNA abundance and protein expression reached peak levels at ∼6 h after bacteria feeding (Figure 3A and 3C). In contrast, peak expression after a *P. berghei*-infected blood meal occurred at ∼24 h (Figure 3A). This difference is likely explained by the timing of the physical interactions between the inducing agent and the midgut epithelium. Following bacteria feeding, the contact between bacteria and the midgut epithelium likely occurs soon after ingestion. However, the ingestion of a blood meal triggers the formation of the peritrophic matrix, a chitinous acellular layer that completely surrounds the blood bolus, physically separating it from the midgut epithelium [25]. Not until *Plasmodium* ookinetes differentiate, traverse the peritrophic matrix, and invade the midgut epithelium are the presumed signals leading to *As*SRPN6 activation initiated.

This interpretation implicating the role of the peritrophic matrix in preventing immune recognition is consistent with the spatial pattern of SRPN6 expression by immunofluorescence. As shown in Figure 3B, bacteria feeding trigger a generalized activation of *As*SRPN6 protein accumulation across the majority of the midgut epithelium. This is in contrast to *Plasmodium* infection, where *As*SRPN6 and *Ag*SRPN6 protein seems to accumulate only in invaded cells [19].

In addition, the mechanistic role of *As*SRPN6 as a putative serine protease inhibitor may differ in its response to bacteria or *Plasmodium* parasites. *As*SRPN6 is detected in both its native form and in a higher molecular weight complex following *E. cloacae* feeding (Figure 3C). These experiments suggest that *As*SRPN6 may form a covalent complex with a serine protease (of ∼25 kDa), similar to the inhibitory complexes formed by other mosquito serpins [26]. This is in contrast to the degraded or processed forms of *As*SRPN6 detected following *Plasmodium* infection [19], and may imply that *As*SRPN6 has alternative functions in the mosquito immune response to different pathogens. Alternatively, these differences could also be explained by digestive enzymes present within the blood meal or by direct interactions with different pathogen-specific proteases. One may speculate that the ∼80 kDa complex that we identified following *E. cloacae* infection is similar to the recombinant *Ag*SRPN6 complexes produced *in vitro* with hemolymph proteases from the lepidopteran insect, *Manduca sexta* [21]. However, further experiments must be performed to identify the target proteinases of *As*SRPN6 and *Ag*SRPN6 *in vivo*.

### *E. cloacae* Inhibits *P. falciparum* Development in *An. stephensi*

**Figure 4:**
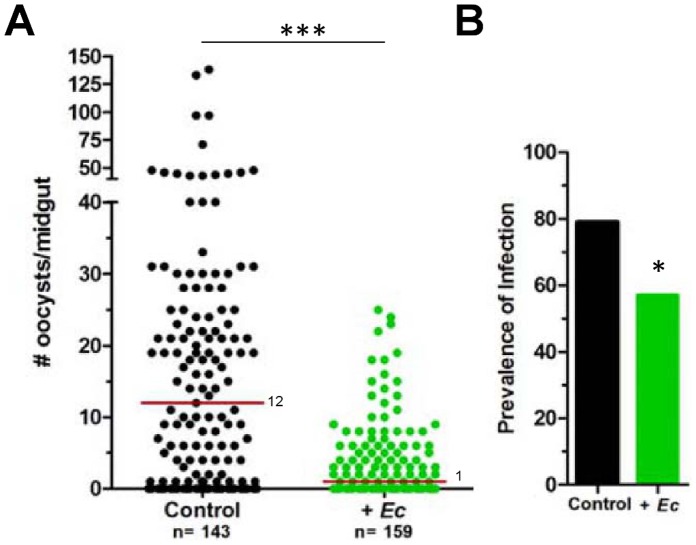
*E. cloacae* inhibits *P. falciparum* development in *An. stephensi*. (A) Mosquitoes were fed on a *P. falciparum* gametocyte culture mixed either with medium alone (control) or with *E. cloacae* (+*Ec*; at a final concentration of 1×10^6^/ml). After 8 days, oocyst numbers were counted by mercurochrome staining of dissected midgut samples and the data were pooled from four independent experiments. Median oocyst numbers are depicted by the red line and the *P*-value was determined using a Mann–Whitney U test. The total numbers (n) of mosquitoes analyzed are denoted below each sample. The percentage of mosquitoes containing at least one *P. falciparum* oocyst (or prevalence of infection) is shown in (B). Samples were analyzed by Chi-squared analysis to determine significance. *P*-values are denoted by asterisks (* = *P*<0.05; *** = *P*<0.001).

Previous reports have demonstrated that the presence of bacteria within the mosquito midgut greatly influences *Plasmodium* development [8], [9], [12], and that field isolates of an *Enterobacter* species have a profound effect on *P. falciparum* development [8]. Our results (Figures 1 and 3) show that *E. cloacae* strongly induces *As*SRPN6 expression (and other components of the mosquito immune response) in the mosquito midgut. To determine the effects of our *Enterobacter* strain on parasite development, we fed *An. stephensi* mosquitoes on a *P. falciparum* gametocyte culture mixed with *E. cloacae* bacteria [8]. The bacteria caused a dramatic decrease in the number of parasites that developed to the oocyst stage, as compared to control mosquitoes (Figure 4A). In addition, there was a significant decrease in prevalence (percent mosquitoes that were infected) that accompanied this reduction in oocyst numbers (Figure 4B). To determine whether this decrease is due to interference of *Plasmodium* development prior to midgut invasion similar to an *Enterobacter* strain described in Cirimotich *et al.* [8], we measured the effect of bacteria on the formation of mature ookinetes. As shown in Table 1, the presence of bacteria up to a concentration of 10^6^/ml had no effect on the development of *P. falciparum* gametocytes to mature ookinetes in the mosquito midgut. These results suggest that *E. cloacae* interferes with ookinete invasion and traversal of the midgut or alternatively, with the differentiation of ookinetes into oocysts. This is in contrast with the pre-invasion phenotype described for a natural *Enterobacter* isolate [8], thus highlighting the differences in the inhibitory mechanisms against *Plasmodium* development even between bacteria of the same genus. However, considering that these experiments were performed in different mosquito species, we cannot rule out that the observed differences are due to differences in how *An. stephensi* and *An. gambiae* respond to bacteria rather than differences between *Enterobacter* species.

**Table 1:** Effect of *E. cloacae* on *P. falciparum* ookinete development in the mosquito.

Experiment	Control	*E. cloacae*/ml		
		10^4^	10^6^	10^8^
1	104±13	99±5	103±23	53±8*
2	190±21	210±11	173±19	67±6*

*An. stephensi* females were fed *P. falciparum* gametocyte cultures mixed with medium alone (control) or with *E. cloacae* at the indicated concentrations. Individual midguts were dissected 20 h later and the number of ookinetes per gut was determined. Each value is the mean number of *P. falciparum* ookinetes (±SD) obtained from five midguts (n = 5) under each experimental condition. Each experiment was individually analyzed using a One-way ANOVA with a Dunnett’s post-test to determine the effects of *E. cloacae* on *P. falciparum* ookinete development. Asterisks denote significant differences with a *P* value of <0.05.

### *As*SRPN6 Contributes to *E. cloacae* Inhibition of *P. falciparum* Development in *An. stephensi*

Bacteria induce a broad spectrum of immune responses in the mosquito midgut to promote resistance to malaria parasites [9], [27], [28]. As shown in Figure 3, *E. cloacae* strongly induce *As*SRPN6 expression in the mosquito midgut. Since SRPN6 activity has previously been implicated in the mosquito anti-*Plasmodium* immune response [19], [20], we wanted to evaluate the contribution of *As*SRPN6 toward this bacterial-induced resistance. To investigate the role of *As*SRPN6 in the bacteria-induced inhibition of *P. falciparum* development, three groups of mosquitoes were fed as follows: (i) naïve mosquitoes fed with an infectious *P. falciparum* gametocyte culture; (ii) dsGFP-injected control mosquitoes fed with the same gametocyte culture mixed with *E. cloacae*; and (iii) dsSRPN6-injected experimental mosquitoes fed with the same gametocyte culture mixed with *E. cloacae*. Group (i) served as a reference, while group (ii) was used to confirm the inhibition of *P. falciparum* by the bacterium (as in Figure 4) and to control for any non-specific effects of dsRNA injection. Group (iii) was to assess the role played by *As*SRPN6, if any, in the inhibition by bacteria.

**Figure 5:**
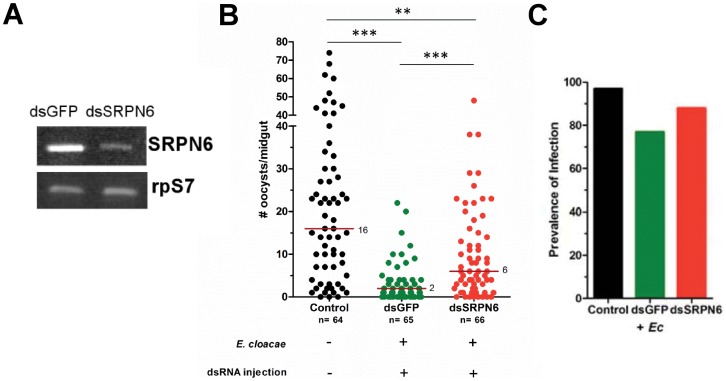
*E. cloacae*-mediated inhibition of *P. falciparum* development is reversed by SRPN6 silencing. (A) Semi-quantitative RT-PCR analysis was used to determine the effects of gene silencing on *An. stephensi* midgut samples after feeding with a *P. falciparum* gametocyte/*E. cloacae* (10^6^/ml) mixture. SRPN6 mRNA abundance in midguts of dsSRPN6-injected mosquitoes was suppressed when compared with dsGFP controls. Ribosomal protein S7 (rpS7) served as a loading control. (B) *An. stephensi* mosquitoes were fed on a *P. falciparum* gametocyte culture (control) or were injected with dsGFP or dsSRPN6 and fed a *P. falciparum* gametocyte/*E. cloacae* (10^6^/ml) mixture. Midgut oocyst numbers were determined after 8 days by staining with mercurochrome. Data were pooled from three independent experiments and analyzed using Kruskal-Wallis analysis and a Dunn’s post-test to determine significance. Median oocyst numbers are depicted by the red line and the total numbers (n) of individual mosquitoes analyzed are denoted below each sample. The presence (+) or absence (−) of *Enterobacter* feeding or dsRNA treatment are shown below each sample. *P*-values are denoted by asterisks (** = *P*<0.01; *** = *P*<0.001). (C) Bar graphs showing the prevalence of infection among samples shows that SRPN6-silencing increase the percentage of mosquitoes containing at least one *P. falciparum* oocyst, although the results are not significant when analyzed by Chi-squared analysis.

The efficiency of dsRNA-mediated silencing of *As*SRPN6 was evaluated by RT-PCR (Figure 5A). Similar to our experiments in Figure 4, the addition of *E. cloacae* significantly inhibited parasite development in both the dsGFP and dsSRPN6 (Figure 5B), although we cannot distinguish between the effects of *E. cloacae* feeding and dsRNA treatment. However, there was a significant increase in the number of *P. falciparum* oocysts that develop in the dsSRPN6 mosquitoes when compared to the dsGFP controls (Figure 5B). Silencing of *As*SRPN6 expression also resulted in an increased prevalence of infection (Figure 5C). This would suggest that the loss of *As*SRPN6 function following dsRNA-silencing partially rescues the inhibitory effects of *E. cloacae* that limit parasite growth. While other immune components are likely triggered by *E. cloacae*, our data suggest that inhibition of parasite development is partially mediated by *As*SRPN6 function.

## Discussion

Midgut invasion is a critical step in the propagation of many pathogens in the mosquito and essential for their transmission. Moreover, during midgut invasion parasite numbers are the lowest of their life cycle in the mosquito, making this stage a prime target for transmission control [1]. In recent years, progress has been made in outlining the mechanisms of the mosquito immune response against *Plasmodium*, but our understanding of this process remains incomplete. Here we report on experiments that provide new insights into the mechanisms of action of the immune protein *As*SRPN6, and the interplay between the mosquito microbiota and the mosquito response to *Plasmodium* infection. Our findings demonstrate that *As*SRPN6 is differentially activated by certain bacteria and that this activation is more efficient when bacteria are exposed to the lumenal surface of the midgut epithelium. In addition, we demonstrate the importance of bacteria in limiting parasite development through the activation of the innate immune response and find that the *E. cloacae-*mediated inhibition of *P. falciparum* development in *An. stephensi* is partially mediated by *As*SRPN6 function.

Mosquito midgut epithelial cells serve as the first point of contact between ingested pathogens and their vector. These cells display an active immune response that limits parasite propagation [9], [27], [29]. Previous observations suggest that SRPN6 is an important component of the mosquito epithelial response to *P. berghei* [19], and this report shows that in addition to *Plasmodium*, *As*SRPN6 is selectively induced primarily by gram-negative bacteria. Our experiments suggest that the gram-negative *E. cloacae* induces the highest levels of *As*SRPN6 transcript, but the specific bacterial elicitors of this response are unknown. Recent work suggests that *Ag*SRPN6 may be induced by physical damage to the midgut epithelium as a result of *P. berghei* ookinete invasion [22], but it is unknown if *E. cloacae* is able to similarly damage the midgut epithelium. Other gram-negative bacteria have been shown to compromise the integrity of the *Drosophila* midgut epithelium through the production of pore-forming toxins, raising the possibility that *As*SRPN6 induction mediated by *E. cloacae* may occur via similar mechanisms [30]. We have recently identified that a LITAF-like transcription factor (LL3) is involved in the transcriptional regulation of SRPN6 in response to *Plasmodium* invasion [22], but further experiments must be performed to determine if LL3 is involved in the regulation of the *As*SRPN6 immune response to *E. cloacae*. It is unclear whether the mechanisms of SRPN6 activation are conserved between bacteria and *Plasmodium* parasites, yet our experiments demonstrate that *As*SRPN6 immune activation by *E. cloacae* leads to cross immune protection against *P. falciparum* as previously described with [9], [31].

To examine the signals that lead to *As*SRPN6 immune activation, we determined that the injection of bacteria into the mosquito hemocoel had no effect on *As*SRPN6 expression in the midgut. *As*SRPN6 expression in the carcass was minimally activated as a result of wounding (physical damage resulting from injection), likely through expression in hemocytes or fat body. These observations suggest that the basal epithelial cell surface does not possess the appropriate sensing mechanisms (i.e. - receptors) or that the basal lamina constitutes a barrier that prevents physical interaction between the bacteria and the basal midgut epithelial cell surface. In addition, it also implies that *As*SRPN6 is not a major component of *An. stephensi* humoral immune response since bacteria introduced into hemocoel activate anti-microbial gene expression in the fat body [27], but not *As*SRPN6. This would suggest that the “natural” evolutionary role of *As*SRPN6 may be to attenuate the growth of endogenous flora and ingested bacteria within the midgut, rather than having a role in the systemic humoral response. Most likely, the required signaling needed for *As*SRPN6 immune activation depends either on direct interactions on the lumenal surface or on intracellular signals produced in response of pathogen invasion.

Our data suggest a mechanism of inhibition that is dependent upon mosquito immune activation, mediated in part, by *As*SRPN6 function. This activation occurs soon after feeding (∼6 h post blood meal peak) much before parasite invasion of the midgut epithelium (∼24 h post blood meal). SRPN6 and other immune genes may be activated through the direct interaction of *E. cloacae* with the midgut epithelium before the onset of ookinete invasion. It has been well documented that the mosquito immune system is capable of being primed by bacterial injection [31], or by the presence of the intracellular symbiont *Wolbachia* to confer resistance to *Plasmodium* development in mosquitoes [32], [33]. Through priming of the immune response by feeding *E. cloacae*, midgut epithelial cells are “loaded” with immune proteins that also confer anti-*Plasmodium* properties, resulting in fewer parasites that are able to successfully develop in the mosquito host.

Data presented here raise the possibility that *As*SRPN6 may be an integral component of the mosquito innate immune response to bacteria, in addition to its previously defined roles in anti-*Plasmodium* immunity [19], [20]. As a predicted serine protease inhibitor, SRPN6 may interact with proteases that regulate mosquito immune signaling pathways [26], [34] or through direct interactions with the pathogen as in other species [35], [36]. Future studies aim to determine the molecular mechanisms leading to SRPN6 activation and understanding how expression of SRPN6 leads to inhibition of *Plasmodium* development by identifying its mode of action.

In summary, our data demonstrate that lumenal exposure to *E. cloacae* mediates an anti-*Plasmodium* response in *An. stephensi* that is partially dependent on *As*SRPN6 function. Moreover, our results support applications that rely on the midgut microbiome of anopheline mosquitoes (natural isolates or genetically engineered) to interfere with the transmission of malaria [8], [37].

## Materials and Methods

### Ethics Statement

This project was carried out in accordance with the recommendations of the Guide for the Care and Use of Laboratory Animals of the National Institutes of Health. The animal protocol was approved by the Animal Care and Use Committee of the Johns Hopkins University (protocol number M009H58). Anonymous human blood used for parasite cultures and mosquito feeding was obtained under IRB protocol NA 00019050 approved by the Johns Hopkins School of Public Health Ethics Committee. Informed consent is not applicable.

### Mosquito Rearing and *Plasmodium* Infections

*An. stephensi* mosquitoes were reared under standard insectary conditions of 27°C and 80% relative humidity and maintained on 10% sucrose. *P. berghei* ANKA 2.34 was maintained and used to infect mosquitoes as previously described [38]. NF54 isolates of *P. falciparum* gametocyte cultures were obtained from the Johns Hopkins Malaria Research Institute Parasite Core facility. The culture was washed and brought up in normal human serum (Interstate Blood Bank, Memphis, TN) plus human RBCs to 45% hematocrit and 0.3% gametocytemia. Infective blood was placed into water-jacketed glass membrane feeders warmed to 37°C. Mosquitoes were allowed to feed for 20 min, and were maintained thereafter for 8 d in an incubator at 26°C and 80% humidity. Only midguts from fully gravid females were analyzed for *Plasmodium* infection by staining with 0.1% mercurochrome to count oocyst numbers.

### Bacteria Feeding

Bacteria were washed in phosphate buffered saline (PBS) and suspended in latex feeding buffer [39] at a final concentration of 1×10^6^ colony forming units (cfu)/ml or as indicated. Adult mosquitoes were maintained from the time of hatching in sterile 10% sucrose solution containing 10 units/ml penicillin and streptomycin. Antibiotic solution was replaced by sterile sugar solution two days prior to the infectious blood meal. Mosquitoes were starved overnight and fed on medium or bacteria by use of water-jacketed glass-membrane feeders warmed to 37°C. Bacteria suspended in PBS at 10^6^ cfu/ml were injected (∼100 nl) into the mosquito hemocoel through the thorax. For mixed gametocyte/bacteria feeding, *E. cloacae* was washed in ookinete medium [40] and then mixed with gametocytes to final 0.3% gametocytemia at the desired bacterial concentration.

### *SRPN6* Gene Expression Analysis

Northern blot analysis was performed as previously described [41]. Total RNA was isolated from 5–20 engorged female mosquito midguts or carcass (all tissues minus the gut) at selected time points following bacteria feeding or a parasite-infected blood meal with TRIzol reagent (Molecular Research Center Inc.). A mosquito mitochondrial rRNA gene was used as a loading control [41].

For quantitative RT-PCR, RNA samples were treated with DNase and reverse-transcribed with Superscript III (Invitrogen) using random hexamer primers. Real-time quantification was performed using the SYBR Green PCR master mix (Applied Biosystems) and ABI Detection System ABI Prism 7000 (Applied Biosystems). All PCR reactions were performed in triplicate. Specificity of the PCR reactions was assessed by analysis of melting curves for each data point. The ribosomal protein S7 gene was used for normalization of cDNA templates as described previously [19].

### Immunofluorescence and Immunoblotting

*An. stephensi* midgut sheets were prepared from buffer-fed or *E. cloacae*-fed mosquitoes 6 h after a bacterial meal and stained by immunofluorescence as previously described [42]. Midgut sheets were incubated with affinity-purified SRPN6 antibody (1∶1000) raised against the full-length *An. gambiae* SRPN6 protein [19] and SRPN6 was detected with Alexa Fluor® 488-labeled goat anti-rabbit secondary antibody (1∶1000, green, Molecular probes). Cell nuclei were stained with DAPI (Roche Applied Science).

For immunoblotting, 10 midgut sheets were prepared from *An. stephensi* mosquitoes fed on an *E. cloacae* bacterial meal were placed in 70 µl of 1×Laemmli buffer and boiled for 5 min. The equivalent of 2 midgut sheets were separated by 12% SDS-PAGE and transferred to a PVDF membrane. The membrane was incubated with affinity-purified anti-SRPN6 antibody (1∶10,000) and the bound antibody was detected with a horseradish peroxidase-linked anti-rabbit IgG (Pierce, 1∶25,000 dilution) by exposing the blots to X-ray films. Membranes were stripped by two 30-minute washes in 100 mM 2-mercaptoethanol, 2% (w/v) SDS, 62.5 mM Tris-HCl, and pH 6.7 at 50°C. The stripped membranes were incubated with actin antibody (1∶500; A2066, Sigma) for assessment of the relative amount of protein analyzed in each lane.

### Detection of *P. falciparum* Ookinetes

To determine the number of midgut ookinetes, mosquito midguts were dissected in PBS 20 h post gametocyte feeding. Each gut was suspended in 100 µl of PBS, homogenized gently and 5 µl of the homogenate was spotted on a glass slide and air dried. *P. falciparum* ookinetes were detected by immunofluorescence as described above using an anti-Pfs25 monoclonal antibody (1∶1000) and detected with Rhodamine red™-X-labeled goat anti-mouse secondary antibody (1∶1000, Molecular probes).

### RNA Interference

A 785 bp (nucleotide 709 to 1693) region of AgSRPN6 cDNA (AGAP009212-RA) was amplified using T7-promoter-flanked primers (F: 5′ T7-AGCCCCAGCTCGAGTGTGGTG-3′ and R: 5′T7-AAATAACGAGACGCGTCAGAAGTA-3′). Double stranded RNA (dsRNA) was synthesized using the MEGAscript RNAi kit (Ambion) and purified according to the manufacturer’s instructions. Approximately 300 nl of dsRNA targeting SRPN6 or GFP (control) solution at 3 µg/µl was injected into adult female mosquitoes using a nanojet injector as previously described [43]. Two days later, mosquitoes were fed on a *P. falciparum* gametocyte culture mixed with *E. cloacae*. To determine silencing, SRPN6 expression was analyzed by semi-quantitative RT-PCR approximately 6 h after an *E. cloacae* bacterial meal.
